# The emerging role of epigenetics in human autoimmune disorders

**DOI:** 10.1186/s13148-019-0632-2

**Published:** 2019-02-26

**Authors:** Roberta Mazzone, Clemens Zwergel, Marco Artico, Samanta Taurone, Massimo Ralli, Antonio Greco, Antonello Mai

**Affiliations:** 1 Department of Chemistry and Technologies of Drugs, Sapienza University of Rome, P.le A. Moro 5, 00185 Rome, Italy; 2 Center for Life Nano Science@Sapienza, Italian Institute of Technology, Viale Regina Elena 291, 00161 Rome, Italy; 3 Department of Sense Organs, Sapienza University of Rome, P.le A. Moro 5, 00185 Rome, Italy; 4 IRCCS G.B. Bietti Foundation, Via Livenza, 3, 00198 Rome, Italy; 5 Pasteur Institute - Cenci Bolognetti Foundation, Sapienza Università di Roma, P.le Aldo Moro 5, 00185 Rome, Italy

**Keywords:** Epigenetics, Gene expression, Autoimmune diseases, Epigenetic pathways

## Abstract

Epigenetic pathways play a pivotal role in the development and function of the immune system. Over the last decade, a growing body of studies has been published out seeking to explain a correlation between epigenetic modifications and the development of autoimmune disorders. Epigenetic changes, such as DNA methylation, histone modifications, and noncoding RNAs, are involved in the pathogenesis of autoimmune diseases mainly by regulating gene expression. This paper reviews the importance of epigenetic alterations during the development of the most prevalent human autoimmune diseases, such as systemic lupus erythematosus (SLE), rheumatoid arthritis (RA), systemic sclerosis (SSc), Sjogren’s syndrome (SS), autoimmune thyroid diseases (AITD), and type 1 diabetes (T1D), aiming to provide new insights in the pathogenesis of autoimmune diseases and the possibility to develop novel therapeutic approaches targeting the epigenome.

## Background

**Fig. 1:**
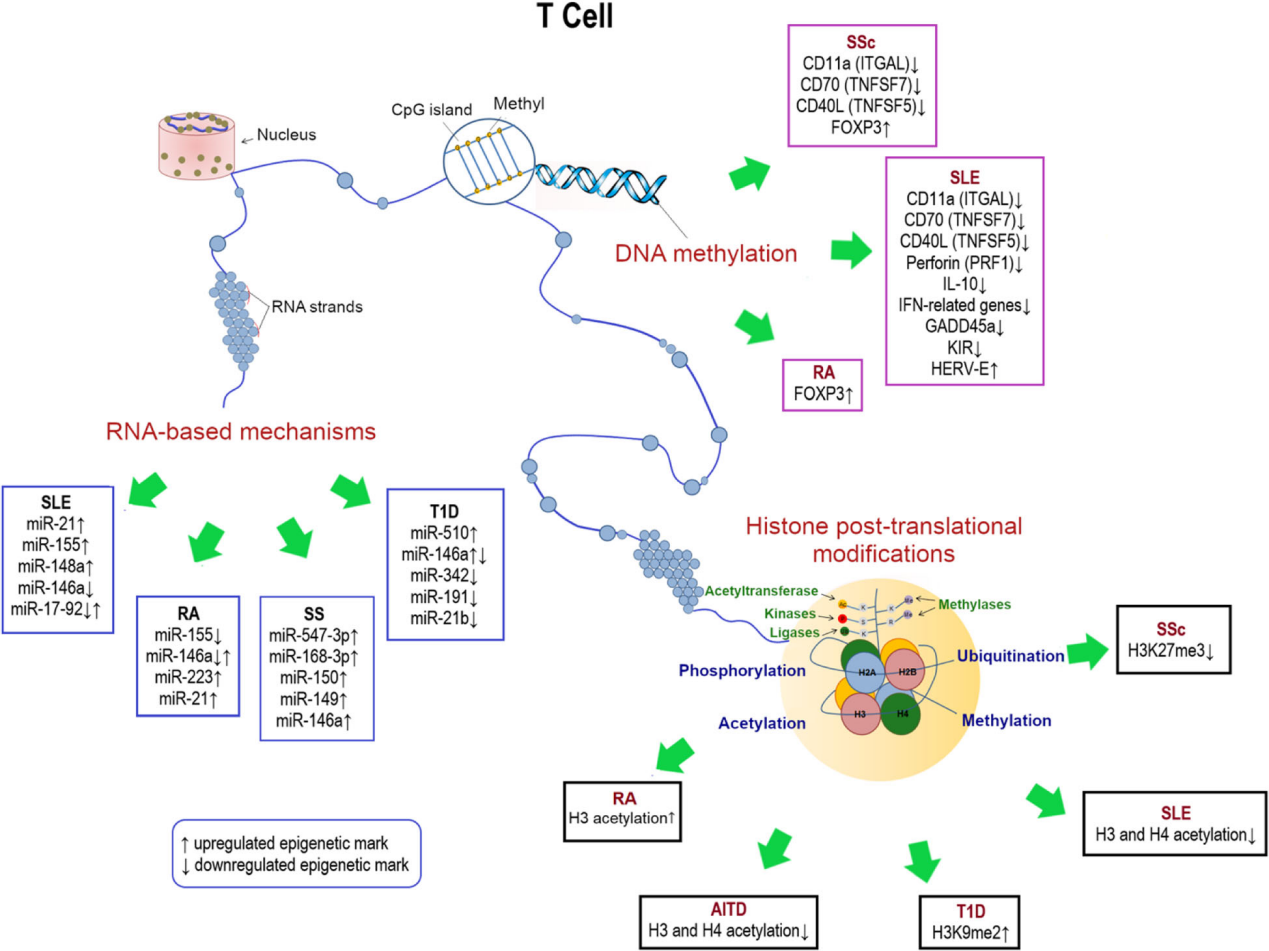
Schematic representation of key epigenetic mechanisms (DNA methylation, histone post-translational modifications, and RNA-based mechanism) involved in the pathogenesis of autoimmune diseases, including SLE, RA, SSc, SS, AITD, and T1D

Epigenetic mechanisms, known for their ability to regulate gene transcription and genomic stability, are key players for maintaining normal cell growth, development, and differentiation [1]. The term “epigenetics” can be outlined as the meiotically/mitotically heritable alterations in gene expression, related to environmental factors, without changes to the sequence of bases in the DNA [1]. Since genome-wide profiling in some cases does not give a sufficient answer to explain the complex biological processes in autoimmune disorders, epigenetic modifications are retained additional regulators in immune responses (Fig. 1). Epigenetic dysregulation directly influences the development of autoimmunity by regulating immune cell functions [2]. The recognition of the complexity of the interaction between epigenetic events and the alteration of the immune system in autoimmune disorders is a prominent challenge for the discovery of novel potential therapeutic strategies. Epigenetic mechanisms, such as DNA methylation, chromatin remodeling, and noncoding RNAs, have been identified as crucial regulators in cellular immunity, owing to their mechanisms in modulating gene expression and transcription in targeted cells and tissues [3]. Extensive evidences indicate that autoimmune diseases are mainly an interplay of genetic and non-genetic factors, although the role of the latter ones often remains unclear. Over the last decade, the influence of epigenetic modifications on innate and adaptive immunity has been intensively investigated, especially in autoimmune disorders.

## Epigenetic mechanisms

### DNA methylation

DNA methylation is one of the most studied epigenetic marks and typically occurs at the pyrimidine C5 position of cytosine residues by transferring a methyl group from *S*-adenosyl-l-methionine (SAM) through the catalytic action of DNA methyltransferases (DNMTs) [4–6]. DNA methylation influences a large variety of biological processes such as transcriptional repression, reversible promoter silencing, and chromosomal instability [7, 8]. The various functions and diverse subtypes of DNMTs have been summarized well in various reviews [9–11].

One of the crucial functions of DNA methylation is the maintenance of the T cell regulation. Recent findings underlined the critical role of DNA methylation in numerous autoimmune diseases by altering gene expression profiles [12–15]. Several factors such as environmental influences, genetic variants, drugs, and miRNAs influencing the DNA methylation status have been linked to autoimmune disorders.

### Histone modifications

Histone-modifying enzymes have an essential role in modulating chromatin compaction state, nucleosomal processes, and DNA repair [16–18]. Some HDAC inhibitors (HDACi) have been widely studied as a means to increase the transcription of various genes, including those involved in autoimmunity [19–21]. Moreover, HDACi have been described to influence immune and inflammatory processes, mainly involving T cells [22, 23]. Acetyl and methyl marks inserted on histones could also interact with the corresponding “reader” proteins to add complexity to this scenario. Some evidence suggested that posttranslational modifications are implicated in the development and regulation of different cell lines and in the modulation of immune tolerance and autoimmune disorders [24, 25].

### Noncoding RNAs

Long noncoding RNAs (lncRNAs) are a huge and diverse class of transcribed RNA molecules with a length of more than 200 nucleotides. Usually, they are capped, polyadenylated, and spliced without having any coding function for proteins [26]. LncRNAs have been described as key players of gene regulation in various human pathologies due to their implication in numerous cellular and biological events regulating heterochromatin formation, histone modifications, DNA methylation targeting, and gene silencing [27, 28]. Furthermore, lncRNA can activate regulatory complexes regulating the development and differentiation of various immune cell types via an active protein expression control. MiRNAs are responsible for the regulation of about 60% of all mRNAs and are implicated in numerous disorders, such as cancers, inflammatory processes, and metabolic diseases [29, 30]. Due to their essential role in the hematopoietic development, as well as cell activation and differentiation, abnormal expression of miRNAs might lead to the onset of autoimmune diseases [31, 32].

## Systemic and organ-specific autoimmune diseases

Autoimmune diseases are characterized by an immune response to antigenic components of the host itself (autoantigens). Two main types of autoimmune diseases can be distinguished: on the one hand, the systemic ones and on the other, the organ-specific ones. In systemic diseases, the immune system attacks in a generalized manner its own antigens in several organs, while in organ-specific diseases the immune response is directed towards a single organ. Examples of systemic and organ-specific autoimmune diseases with known autoantigen targets are illustrated in Table 1.

**Table 1:** Most relevant autoimmune diseases with known autoantigen targets

Systemic autoimmune diseases					
Disease	Organ	Autoantigens	Mechanism of damage	Worldwide prevalence (%)	Ref.
Rheumatoid arthritis	Joints, lung, heart, etc.	IgG, filaggrin, fibrin etc.	T cell in joint/antibody	0.8	[33–35, 72–74]
Systemic lupus	Skin, joints, kidneys, brain, lungs, heart, others	Nuclear antigens (DNA, histones, ribonucleoproteins), others	Antibody	0.1	[33–35, 49, 50]
Polymyositis/dermatomyositis	Skeletal muscle (predominant), lungs, heart, joints, others	Muscle antigens, aminoacyl-tRNA synthetases, other nuclear antigens	T cell/antibody	< 0.01	[33–36, 95]
Systemic sclerosis	Lungs, river, kidneys, heart, skin, etc.	Dermal fibroblast antigens, fibrillarin-1, metalloproteinases, etc.	Antibody	0.3	[33–35, 94, 95]
Sjogren’s syndrome	Connective tissue, salivary gland, lungs, bowel, etc.	Nuclear antigens, carbonic anhydrase I (CA-I), profiling	T cell/Antibody	0.1–0.6	[33–35, 115, 116]
Organ-specific autoimmune diseases					
Disease	Organ	Autoantigens	Mechanism of damage	Prevalence (%)	Ref.
Thyroiditis (autoimmune)	Thyroid	Thyroglobulin, thyroid peroxidase	T cell/antibody	1.0–2.0	[33, 35, 130–134]
Gastritis	Stomach	H^+^/K^+^ ATPase, intrinsic factor	T cell/antibody	1–2 in > 60 years old	[33, 35]
Celiac disease	Small bowel	Transglutaminase	T cell/antibody	0.2–1.1	[33, 35]
Graves’ disease	Thyroid	Thyroid-stimulating hormone receptor	Antibody	0.2–1.1	[33, 35, 130, 131]
Vitiligo	Melanocytes	Tyrosinase, tyrosinase-related protein-2	T cell/antibody	0.4	[33, 35]
Type 1 diabetes	Pancreas β cells	Insulin, glutamic acid decarboxylase	T cell	0.2–0.4	[33, 35, 149–152]
Multiple sclerosis	Brain, spinal cord	Myelin basic protein, proteolipid protein	T cell	0.01–0.15	[33, 35]
Hepatitis (autoimmune)	Liver	Hepatocyte antigens (cytochrome P450)	T cell/antibody	< 0.01	[33, 35]
Myasthenia gravis	Muscle	Acetylcholine receptor	Antibody	< 0.01	[33, 35]
Primary biliary cirrhosis	Liver bile ducts	2-oxoacid dehydrogenase complexes	T cell/antibody	< 0.01	[33, 35]
Pemphigus	Skin	Desmogleins	Antibody	< 0.01–> 3.0	[33, 35]

## Systemic autoimmune rheumatic diseases (SARDs)

Systemic autoimmune rheumatic diseases (SARDs) belong to a group of rare chronic inflammatory conditions including rheumatoid arthritis (RA), systemic lupus erythematosus (SLE), systemic sclerosis (SSc), and Sjogren’s syndrome (SS). Autoimmunity is defined by the breakdown of self-tolerance that produces a state of abnormal humoral and cell-mediated responses against self-components. Collectively, SARDs affect up to 5% of the world population and, in particular, people of work-force age [33, 34]. Until now, no effective treatments have been identified for SARDs, even though the use of glucocorticoids has been considered as a first-line therapy. Nowadays, however, antimalarial and immunosuppressive drugs are most commonly used due to their limited long-term side effects. Such autoimmune disorders are often associated with an autoimmune dysregulation which determines morbidity and, in most cases, premature mortality [35, 36]. In particular, most of these conditions happen when the immune system produces autoantibodies (ANA) directed against intracellular antigens. So, ANA are considered as convincing serological hallmarks of SARDs, which are routinely detected via an indirect immunofluorescence (IF) assay [37]. Although many progresses have been performed in the last 15 years, there is an unmet need to find an innovative and successful therapy to fight SARDs, especially RA, the most prevalent autoimmune disease. Understanding the molecular mechanisms of SARDs appears to be extremely important to achieve beneficial outcomes in these chronic conditions. However, similar biologic pathways that underlie SARDs in RA, SLE, and SSc may suggest novel mechanistic similarities. Indeed, these three chronic diseases have in common demographic distribution (the most affected individuals are women), some signs and symptoms (arthritis, lung and vascular disease), serological elements (ANA and anti-Ro52/TRIM21 antibodies), immunological components (type I interferon signature and complex abnormalities in CD4C T lymphocyte function, in particular Th17 and Treg cell subsets), and genetic similarities (e.g., MHC class II alleles, IRF5, STAT4, PTPN22 loci) [38–44]. More and more studies demonstrated that epigenetically altered immune components, such as CD4^+^ T cells and costimulatory molecules, are key drivers of SARDs, thereby compromising cellular immune system function and regulation [45–48]. Characterization of epigenetic modifications that occur across these autoimmune diseases may yield valuable insights into their pathogenesis and treatment. Thus, in an attempt to determine the most important epigenetic changes in SARDs, researchers investigated the role of epigenetic processes in regulating autoimmunity. For many years, epigenetic implications for the most important related forms of autoimmunity including RA, SLE, SSc, and SS have been studied in order to find a tight association between epigenetics and systemic autoimmune, providing novel regulatory mechanisms for SARDs (Table 2).

**Table 2:** Epigenetic changes in systemic lupus erythematosus (SLE), rheumatoid arthritis (RA), systemic sclerosis (SSc) and Sjogren’s syndrome (SS)

Autoimmune disease	DNA methylation	Histone modification	miRNA
SLE	↓ CD70, ↓ CD11a, ↓ CD40L, ↓ perforin ↓ T cell ↓ IFN signature [50–63]	↑ H3, H4 methylation ↑ H3, H4 acetylation [64–67]	↑ miR-21, ↑ miR-148a [68–71]
RA	↓ T cell ↑ Synoviocytes [79, 80]	↑ H3 acetylation [82–88]	↑ miR-146a, strong link between many microRNAs and DNA methylation [90–93]
SSc	↓ T cell ↓ Fibroblasts ↑ Wnt pathway genes [96–98, 102, 103]	Fibroblasts:↓ H3, H4 acetylation [104, 107, 108]	↑ miR-29a, ↑ miR-196a [109–114]
SS	↓ Type-I IFN pathway genes ↓ T cells [115–121]	No large-scale analysis	↑ miR-146a [122]

### Systemic lupus erythematosus (SLE)

SLE is the most studied autoimmune disease correlated with epigenetic modification. It especially occurs in women and is mainly caused by dysregulation of T lymphocytes, making the disorder complicated and hard to handle. SLE is a chronic autoimmune dysfunction characterized by the development of autoantibodies against nuclear antigens affecting any organ system and tissue, such as kidney and blood vessels. In a recent study, neutrophils and granulocytes from patients with SLE have been described as totally hypomethylated, especially at the gene locus of the interferons *MX1* and *IFI44L* [49]. Discrepancies in monozygotic twins propone environmental factors as crucial drivers for the development of SLE. Epigenetic alterations such as DNA methylation and histone modifications have been found to be able to regulate gene expression in mature T cells. Numerous genes such as CD11a (*ITGAL*), perforin (*PRF1*), CD70 (*TNFSF7*), and CD40LG (*TNFSF5*) in T lymphocytes of SLE patients were found to be hypomethylated [50]. Early studies in SLE CD4^+^ T cells demonstrated a conversion to autoreactivity and an induced lupus-like syndrome after treatment with DNMT inhibitors [51–53]. The auto-reactivity has been shown only in cloned and polyclonal human and murine CD4^+^ cells, but not in CD8^+^ cells, the reason is still undetermined [53, 54]. However, there is strong evidence that auto-reactivity development is concomitant with an increased expression of the adhesion molecules LFA-1 (CD11a/CD18), due to elevated levels of CD11a (*ITGAL*) transcripts. *ITGAL* is an integrin responsible for costimulation and cellular adhesion. The upstream promoter of *ITGAL* can be found to be demethylated in SLE patients’ CD4^+^ cells and depending on the disease activity, and progression CD11a can be found more or less overexpressed [55]. The methylation status of other specific genes has been linked with SLE pathogenesis and development. CD40L, a type II transmembrane protein encoded on the X chromosome by *CD40LG* and functioning as a costimulatory molecule, has been found to be overexpressed in human SLE patients. It still remains elusive why SLE predominantly affects women. *CD40LG* methylation patterns have been associated with female susceptibility to this disease [56]. Following treatment with DNMT inhibitors (azacytidine, procainamide) or ERK pathway inhibitors (hydralazine, PD98059), demethylated *CD40LG* led to induced T cell autoreactivity in vitro [56]. Overexpressed E4BP4 (*NFIL3*), an important human transcription factor, led to the downregulation of the autoimmune responses in SLE patients through inhibiting CD40L expression [57]. Another interesting costimulatory molecule is CD70, encoded by *TNFSF7*, a ligand of CD27. Disruption of CD70-CD27 interaction via blocking antibody allows attenuating lectin-stimulated B cells’ IgG production in vitro [58]. Overexpression in a mouse model can be caused both by traditional demethylating agents and medications associated with drug-induced lupus, including hydralazine and procainamide [59]. Overexpression of CD11a and CD70 in CD4^+^ cells after histone methyltransferase SUV39H1 recruitment resulted in decreased regulatory factor X1 (*RFX1*) levels are important for the determination of CD4^+^ T cell auto-reactivity [60, 61]. Sunahori et al. could demonstrate that inhibiting the catalytic subunit of protein phosphatase 2A (PP2Ac) increased MEK/ERK phosphorylation levels and elevated DNA methylation levels as well as diminished CD70 gene expression [62]. Perforin (*PRF1*), a key regulator gene in cytotoxic CD8^+^ and NK cells, produces a cytolytic protein allowing the disruption and lysis of target cellular membranes [63]. *PRF1* overexpression in both CD4^+^ and CD8^+^ cells, mediated by the gene promoting methylation state, has been linked to SLE. Overexpression of *IL10* in T cells from SLE patients is modulated by abnormal STAT3 activation through the histone acetyltransferase p300 leading to an increase of specific autoantibody production and tissue damage [64]. Another important interleukin, *IL13*, has been considered to play a pivotal role in the development of both human and murine lupus via Th2 cell differentiation [65]. Histone modification pattern in lupus has been less investigated than DNA methylation. A heavily methylated HDAC6 promoter resulted in lower HDAC6 mRNA expression in SLE patients if compared to healthy controls [66]. MRL*lpr* lupus-prone mouse splenocytes showed increased methylation as well as decreased acetylation of histones H3 and H4 compared to control mice; treatment with HDAC inhibitors (HDACi) normalizes aberrant gene expression thus reducing disease activity. However, when lupus T cells are treated by HDACi, including trichostatin A (TSA) and suberoylanilide hydroxamic acid (SAHA), alterations of acetylation levels of acid nuclear transport proteins, transcription factors, and cytoskeleton proteins have been reported [67]. Thus, no valid evidence has been found yet to connect the histone modifications with SLE activity. Several recent studies investigated the role of lncRNAs in lupus pathogenesis. MiR-21, miR-148a, and miR126 are three microRNAs regulated by methylation that are matched with a decreased expression of DNMTs in CD4^+^ T cells of SLE [68]. MiR-148a elicited the expression of CD70 and CD11a, similar to lupus patients [69]. Further, overexpressed miR-155 has been found in Treg cell of MRL*lpr* mice. Its T cell distribution regulating activity has been proven in miR-155 deficient mice which display reduced serum levels of *IL4* and *IL17A*, two specific cytokines secreted by Th2 and Th17 cells, respectively [70]. Another study describes increased serum levels of RANTES in SLE patients harboring, at the same time, lowered MiR-125a expression [71].

### Rheumatoid arthritis (RA)

RA is a chronic and debilitating inflammatory sickness causing destructive arthritis with diffuse damages to joints. Epigenetic mechanisms involved in RA include altered methylation stati in T and B cells as well as in synovial fibroblasts [72–74]. Early methylation studies revealed that patients’ T cells showed a remarkable phenotype, similar to SLE, characterized by global hypomethylation [75, 76]. In particular, methotrexate (MTX), used to treat RA, led to the accumulation of protective Treg cells by inducing FOXP3 expression through the promoter demethylation. However, other secondary effects of MTX, which is namely a folate antagonist, on the RA evolution cannot yet be ruled out. Further studies have shown that MTX can reverse the hypomethylated status in peripheral blood mononuclear cells (PBMC) [77, 78]. In 2012, Nakano and colleagues reported the first epigenome-wide study comparing RA and osteoarthritis (OA) FLS cell lines: out of 1859 differently methylated loci, around 60% were identified as hypermethylated [79]. Thanks to a second recent genome-wide study by de La Rica et al., novel target genes have been discovered as being differently methylated, including *IL6R*, *CAPN8*, and *DPP4*. Comparing rheumatoid arthritis synovial fibroblast (RASF) DNA methylation with miRNA expression and RASF transcriptome data from the Gene Expression Omnibus (GEO), more than 200 of the 714 genes identified had an inverse expression. Moreover, several CpG sites have been detected to be hypermethylated with concomitantly reduced miRNA expression [80]. Histone modifications associated with RA have been less studied; although, an involvement of these epigenetic changes has been documented in RA pathogenesis. Dysregulated HDAC activity in RA PBMCs was not affected by conventional anti-TNF therapies whereas treatment with MI192, a HDAC2/3-selective inhibitor, reduced IL6 production in a dose-dependent manner, thus providing a novel therapeutic approach for RA [81]. Wendling et al. evaluated the relationship between Sirt1 activity/expression and IL-3 levels in PBMCs. High levels of serum IL-3 associated with decreased Sirt1 expression and increased apoptosis in patients with RA have been observed [82]. Kawabata et al. reported an increased HDAC1 expression by TNF-α supplementation in RA synovial fibroblasts [83–85]. HDACi showed anti-inflammatory properties in FLS attenuating disease in animal models of RA. An interesting small open-label trial of givinostat (a HDACi) in a similar disorder, juvenile idiopathic arthritis, outlined improvement in arthritis and a wide safety profile [86]. In RA synovial tissues, the equilibrium between HATs and HDACs activity is heavily disturbed owing to the soaring of HAT activity resulting in hyperacetylation [87]. An increase in *IL6* expression by hyperacetylation of histone H3 has been found in synovial fibroblasts [88]. Ahmed et al. demonstrated that largazole, a marine-derived class I-selective HDACi, provokes the suppression of the TNFα-induced expression of the intracellular adhesion molecule-1 (ICAM-1) and the vascular adhesion molecule-1 (VCAM-1) in RASF, as well as in the reduction of the TNFα-induced MMP2 activity. Additionally, largazole was shown to modulate expression levels of HDAC1, HDAC5, and HDAC6. Of particular interest is the role of HDAC6 in largazole-induced changes of ICAM-1 and VCAM-1 expression levels [89]. Studies trying to explain the effects of microRNAs on RA pathogenesis are emerging more and more in the recent literature. The upregulation of miR-146a with TNF-α and at the same time the downregulation of miR-363 and miR-498 has been found in CD4^+^ cells of RA patients [90]. Despite these pieces of evidence, various studies have shown that miR-146a and miR-155 were decreased in Treg cells after T cell stimulation in RA patients [91]. Additionally, the expression of miR-126a in RA turned out to be elevated, leading to hypomethylated promotors of CD11a and CD70 which in turn led to their overexpression [92]. Further, the increased expression of miR-21 resulted in Treg cell accumulation in synovial fibroblasts of patients suffering from RA [93].

### Systemic sclerosis (SSc)

SSc is a rare and poorly understood autoimmune disease of the connective tissue leading to excessive collagen deposition in the skin and other organs often with a lethal outcome. Aberrant activation of fibroblasts and collagen secretion in SSc conduces to fibrosis. Like SLE, early studies have shown a link to T cell dysfunction and, in particular, autoreactive T cell transfer signals to surrounding fibroblasts inducing the deposit of collagen and initiation of fibrosis [94]. The hypomethylation of CD4^+^ cells, caused at least in part by the downregulation of DNMTs, determined the overexpression of several genes involved in disease progression [95]. Notably, the downregulation of functional demethylating enzymes such as DNMT1, MBD3, and MBD4 resulted in lower levels of methylation at the promoter sites of sensitive genes implicated in SS leading to the overexpression of *CD40L*, *CD11a*, and *CD70* as a relevant feature of SSc [96–98]. The overexpression of the adaptive immune costimulatory molecule *CD40L*, which is crucial for the integrative role in fibrosis of SSc, is a clear sign of the disease in plasma and skin fibroblasts [99, 100]. Furthermore, abrogation of *CD40/CD40L* interaction reduces fibrosis in a SSc mouse model [101]. Recently, other pathways related to SSc have been discussed with particular emphasis on their epigenetic dysregulation mechanisms. One such pathway, Wnt, increases skin fibrosis in SSc patients [102]. Importantly, *Dickkopf-1* and *SFRP-1* are both hypermethylated in dermal fibroblasts and PBMCs in a mouse model of SSc. Thus, treatment with 5-azacytidine reduces Wnt signaling and deletes the fibrosis phenotype [103]. Likewise, inactivation of the collagen suppressor gene *FLI1* by epigenetic hypermethylation reduces the expression of type I collagen in fibroblasts from SSc patients [104]. As aforementioned, fibroblasts play a pivotal role in SSc pathogenesis due to collagen and extracellular matrix component overproduction. As a consequence, various cytokines may be produced to dysregulated immune cells further [105]. Reduced H3K27me3 levels were observed as an important posttranslational modification of CD4^+^ cells in SSc leading to the accumulation of the jumonij demethylase JMJD3 in the aforementioned cells [106]. Similarly, to SLE, several histone modifications have been noted in SSc. Both H3 and H4 acetylation is reduced in SSc fibroblasts [104]. Indeed, the reduced expression of collagen in cultured SSc fibroblasts, as well as fibrosis in animal models, have been reported following HDACi treatment [107, 108]. MicroRNAs have been implicated in SSc concerning to dysregulated fibrosis. In details, miR-29a is decreased in SSc fibroblasts leading to a reduction in Co1 1 and 3 expressions [109]. MiR-29a can avoid pulmonary fibrosis and its knock-down increases pro-fibrotic TGF-β and PDGF-B level. [110]. MiR-21 is regulated by TGF- β and targets smad-7, a key profibrotic gene [111, 112]. At last, miR-155 mediates fibrosis by recruiting keratinocyte growth factor, and overexpressed miR-196 reduces collagen levels by regulating collagen 1 [113, 114].

### Sjogren’s syndrome (SS)

SS is an autoimmune disease affecting salivary and tear glands of unknown origin resulting in eye and mouth dryness. SS is a quite prevalent autoimmune disorder affecting about four million people in the USA. This autoimmune illness is associated with autoantibody production in the blood that is directed against various tissues of the body leading to an inflammatory response. Similarly to other autoimmune diseases, reduced DNA methylation of immune cells is one of the most studied epigenetic marks in SS. Yin et al. found that the T cell costimulatory gene CD70 overexpression on CD4^+^ T cells is due to the hypomethylation of CD70 promoterin SS patients [115]. By contrast, lower expression levels of FOXP3 in SS CD4^+^ T cells have been correlated with DNA hypermethylation [116]. A genome-wide DNA methylation study in naïve CD4^+^CD45RA^+^ cells resulted in the identification of 553 methylated CpG sites. *LTA*, encoding lymphotoxin α, has been found overexpressed in both salivary gland tissue and sera of SS patients [117]. Therefore, its downregulation in the salivary gland of SS mouse model prevents the development of SS. Next, a broader comprehensive study of genome-wide DNA methylation patterns was carried out in whole blood, peripheral CD19^+^ B cells, and minor salivary glands [118]. Differentially hypomethylated sites were identified in type I IFN-induced genes such as *MX1*, *IFI44L*, *PARP9*, and *IFITM1*. The hypomethylation of their promoter in SS B cells was linked to elevated mRNA expression levels. Several other genes have been noted to be differentially methylated, including hypomethylated *STAT1*, *IFI44L*, *IFITM1*, and *USP8*. Conversely, the *RUNX1* gene, an important transcription factor involved in T cell development, was hypermethylated among SS patients. Due to reduced expression of DNMT1 and an increase in GADD45α, epithelial cells from the salivary gland (SGEC) showed global hypomethylation in SS patients. This finding might be associated with infiltrating B lymphocytes. Increased DNA methylation levels in SGEC from SS patients have been reported after the administration of the anti-CD20 monoclonal antibody rituximab [119]. Konsta et al. showed that reduced DNA methylation levels in minor salivary glands might be correlated with overexpression of the epithelial protein cytokeratin-19 (gene *KRT19*) in glandular acini, while high methylation levels resulted in low levels of this protein proven by immunohistochemical analyses [120]. A follow-up study of the same authors proved their concept as the treatment of a human salivary gland cell line with the DNMT inhibitor 5-azacytidine led to *KRT19* mRNA and cytokeratin-19 protein overexpression [121]. To sum up, altered DNA methylation patterns in SGEC may be an important factor in SS pathophysiology probably at least partially via the controlling of the *KRT19*/cytokeratin-19 expression. Actually, no large-scale studies of histone modifications as well as of lncRNAs have been carried out in SS. Increased miR-146a expression has been observed in PBMCs during the onset of SS [122].

## Organ-specific autoimmune diseases

Organ-specific autoimmune diseases, as suggested by the name, are immune response attacks of the body towards healthy cells of a specific organ system occurring as a result of either genetic predisposition or environmental influences or a mixture of both. Many efforts have been performed to understand the inherited autoimmune responses [123]. A large number of polymorphic genes are key players in the genesis of these chronic autoimmune disorders. To date, most details in these processes still need to be understood better even though a large number these genes are apparently involved in setting a threshold for an immune response. Organ-specific autoimmune diseases are usually related to specific human leukocyte class II antigens [124]. In many cases, specific antibodies may be found in patients with chronic and apparently “idiopathic” organ-specific autoimmune diseases. Basically, these antibodies bind to self-antigens in the organ cells or directly on cells, thus leading to their destruction [125]. Examples are the autoimmune thyroid diseases (AITD) and type 1 diabetes (T1D), in which ANA attack the thyroid gland and the immune system which compromises the pancreas, respectively [126, 127]. Normally, the regulation of the autoimmune process involves antigen-specific regulatory cells.

Anti-inflammatory cytokines such as IL-10 and TGF-β are also involved. Up to now, blockers of the immune response produced a greater success in the clinical use than treatments exploiting natural immune regulation. In fact, blocking the immune response is crucial in autoimmunity, even though immunosuppression leads to various side effects, including the reactivation of latent infections and the reduction of immunosurveillance. Thus, antigen-specific immune therapeutical options, instead of rather unspecific therapies targeting the immune system, are an important goal to reach towards the treatment of autoimmune disorders. Over the last decade, scientists figured out that epigenetic changes are clearly correlated with organ-specific autoimmune diseases (Table 3). With the aim to better elucidate mechanisms behind these organ-specific diseases, several studies have been carried out using approved epi-drugs [128, 129] (Table 4). Importantly, the discovery of an epigenetic therapy to treat such autoimmune disorders may unearth potential biomarkers for disease diagnosis and prediction.

**Table 3:** Main epigenetic modifications in autoimmune thyroid diseases (AITDs) and type 1 diabetes (T1D)

Autoimmune disease	DNA methylation	Histone modification	miRNA
AITD	↑ T cells [133–135]	↓ H3, H4 acetylation IFN-α enhances H3K4 methylation [24, 134, 136, 137]	↓ miR-155-5p, ↓ miR-146a-5p, ↓ miR-125a-3p ↓ miR-197a-3p Fibroblasts: ↑ miR-21-5p [138–141]
T1D	↑ T cell ↑ FOXP3 [150, 156, 157]	↑ H3 acetylation [159]	↑ miR-510 ↓ miR-342, ↓ miR-191 [160]

**Table 4:** Effects of Epi-drugs on autoimmune disorders discussed in this review

Autoimmune disorder	Epi-drug	Chemical structure	Target specificity	Autoimmune pathways involved	Ref.
SLE, SSc, SS	Azacytidine (5-AZA) FDA approved, 2004		DNMTi	LFA-1 (CD11a/CD18) ITGAL, IL-6, Wnt pathway, SFRP-1, Dickkoft-1, KRT19, cytokeratin-19 protein	[56, 103, 121]
SLE	Procainamide		DNMT1i	CD70, LFA-1, IL-6	[56, 59]
SLE	Hydralazine		DNMTi	CD70 and other costimulatory proteins	[56, 59]
SLE, T1D	Trichostatin A (TSA)		Pan HDACi	Acid nuclear transport proteins, cytoskeleton proteins, IFN-γ production, transcription activity of Tbx21 in T lymphocytes	[67, 159]
SLE	Vorinostat (SAHA) FDA approved, 2006		Pan HDACi	Acid nuclear transport proteins, cytoskeleton proteins	[67]
RA	Givinostat (ITF2357)		Pan HDACi	Arthritic components, T cells	[86]
RA	Largazole		Class I HDACi	ICAM-1, VCAM-1, MMP2, TNFα, p38, AKT pathway	[89]
RA	Romidepsin (FK228, Depsipeptide) FDA approved, 2009		Class I HDACi	TNFα, IL-1β, p16LNK4a, p21 (WAF1/Cip1)	[84]
RA	MI192		HDAC2/3 inhibitor	IL6	[81]

### Autoimmune thyroid diseases (AITDs)

AITDs are a form of autoimmune diseases mediated by B and T cells. The two main clinical manifestations of AITDs are Flajani-Basedow-Graves’ disease or Graves’ disease (GD) and Hashimoto’s thyroiditis (HT) [130, 131]. HT led to hypothyroidism by cell-mediated autoimmune destruction, while in GD specific autoantibodies against the thyroid stimulation hormone receptor result in hyperthyroidism [132]. Recent literature shed light on the epigenetic mechanisms involved in the pathogenesis of AITD. DNA methylation, histone modifications, and lncRNA have been deeply analyzed in AITDs. However, the clinical utility of epigenetic modulation still remains elusive. Recent findings confirmed that DNA methylation is also in AITDs setting a very crucial epigenetic mechanism. Global DNA hypomethylation was observed in AITD patients, which may result in overexpressed genes important for a correct immune function, or for the activation of immune cells, ultimately leading into an autoimmune attack towards thyroid tissues [133, 134]. In a genome-wide study, Cai et al. have detected in GD patients more than 200 hyper- and hypo-methylated genetic regions, such as *ICAM1*, which partly controls cell antigen processing and presentation; *DNMT1*; and *MECP2* genes [133]. Via epigenetic profiling in CD4^+^ and CD8^+^ cells from GD patients, hypermethylated gene loci of *ICAM1*, *CD247*, and *CTLA4* associated with T cell receptor signaling were observed. [133, 134]. Furthermore, hypermethylation of the first intron area in the *TSHR* gene confirmed that methylation is involved in the development of AITDs. However, DNA hypomethylation levels and susceptibility to AITDs have been correlated to the presence of genetic polymorphisms of DNA methylation-regulatory genes, such as *DNMT1* or methionine synthase reductase (*MTRR*) [135]. Histone modifications play a key role in AITDs, but the exact mechanism(s) in modulating immune tolerance in AITDs is still not fully elucidated. Yan et al. described higher levels of HDAC1 and HDAC2 mRNAs in GD patients thus histone H4 acetylation levels in peripheral blood mononuclear cells of GD patients were lower if compared to those observed in healthy control patient. These results underline the potential importance of aberrant histone modifications in GD patients [24]. In patients with GD, CD4^+^ and CD8^+^ cells were presented higher levels of H3K4me3 and H3K27Ac histone marks [134]. IFN-α, an important cytokine secreted during viral infections, has been identified to be able to lead to higher mono and trimethylation levels of H3K4m in thyroid cells [136]. As previously seen for DNA methylation, genetic polymorphism of histone-modifying genes may result in various malfunctions, and/or other further aberrant histone modifications. Sarumaru et al. demonstrated that rs3758391 and rs4746720 in the SIRT1 gene were linked to higher titers of autoantibodies in AITD affected patients [137]. As previously illustrated for other autoimmune disorders, microRNAs actively modulate in various circumstances the differentiation or activation of immune cells and immune response. Two of the most studied miRNA are miR-155-5p and miR-146a-5p, whose overexpression is believed to break immune tolerance thus fostering the development of autoimmune diseases. For example, GD and HT patients exhibit markedly lower levels of miR-155-5p and miR-146a-5p in the thyroid tissues [138]. Although many research’s efforts showed the potential role of other microRNAs, such as miR-125a-3p, miR-197a-3p, miR-22-3p, and miR-183-5p, only a small number of studies investigated their clinical relevance as diagnostic biomarkers [139–141]. Another epigenetic process called X chromosome inactivation (XCI) has also been discussed in correlation with AITDs [142–144]. X chromosomes are randomly inactivated in females resulting in transcriptional silencing of one of the X chromosomes [145, 146]. Indeed, AITDs are more often observed in females, confirming the compelling role of XCI in these autoimmune disorders. Brix et al. demonstrated that skewed X chromosome inactivation in female twins with GD and HT was significantly higher than in the control populations, suggesting a probable role of XCI in the etiology of AITD [147]. However, Ishido et al. reported no apparent difference between AITD cases and controls. By contrast, a notable relationship between skewed XCI inactivation and the prognosis of GD and HT was observed [148].

### Type 1 diabetes (T1D)

T1D is a chronic autoimmune disease that involves β cell destruction together with a strong inflammatory response. The incidence of diabetes is on the rise worldwide becoming one of the major causes of death. In more detail, the body’s own immune system is destroying the insulin-producing pancreatic β cells first lowering and ultimately ceasing the ability of the pancreas to produce insulin. To date, the disease cannot be cured, although exogenous insulin therapy still remains a life-saving therapy. The destructive process causing T1D is thought to have a pertinent adaptive autoimmune component. In fact, persuasive shreds of evidence suggested that aberrant epigenetic modifications are involved in T1D pathogenesis. The epigenetic involvement in T1D has been nicely reviewed recently [149]. However, environmental factors, in different models of diabetes, influence epigenetic changes and consequently contribute significantly to altered gene expression relevant in T1D development and progression. Over the last decade, some studies have reported that DNA methylation, histone modifications, and noncoding RNAs are considered to have a crucial role in T1D. Again, a genome-wide DNA methylation identified SNP-CpG methylation patterns as potentially relevant for the genetic association of insulin expression and/or secretion in human pancreatic islets [150]. In more detail, candidate genes, such as *GPX7*, *GSTT1*, and *SNX19*, are known to possess a direct influence on various biological processes regarding proliferation and apoptosis in pancreatic β cells. Another study in mouse models also confirmed the implication of epigenetic changes in insulin secretion and diabetes risk [151]. Patients with T1D, compared to the healthy controls, exhibited cell-type-specific gene regulatory circuits crucial for immune cell metabolism and the cell cycle. The latter includes mTOR signaling pathway implicated in the development of diabetes-associated damage [152]. It is believed that epigenetic alteration in diabetes might be the cause for an increased risk for the development and progression of vascular complications. Very likely, these effects are mediated through histone methyltransferases which boost the pro-inflammatory networks implicated in vascular injury [153–155]. Hypermethylated regions of FOXP3 in CD4^+^ cells in T1D resulted in decreased FOXP3 expression and reduced production of regulatory T cells [156]. Additionally, Wang et al. supported the idea of the FOXP3 involvement as its gene hypermethylation was induced by Toll-like receptor 9 (TLR9) in association with a reduced binding activity of interferon regulator factor 7 (IRF-7) [157]. About histone modifications, increased H3K9me2 levels in the promoter of CLTA4, a T1D susceptibility gene, has been correlated with T cell activation [158]. Moreover, Patel et al. suggested that TSA, a well-known HDACi, excites IFN-γ production and increases the transcription activity of Tbx21 in T lymphocytes, alleviating the inflammatory damage of islets [159]. Since T1D shows a broad range of miRNA expression profiles, some miRNAs have been associated with T1D. Upregulated miR-510, as well as the downregulation of miR-342 and miR-191, have been described in T1D. Furthermore, once Treg cells are compared with other types of effector T cells, data showed a substantial change in the expression patterns of miR-146a in conjugation with a decreased expression of eight targeting miRNAs (20b, 31, 99a, 100, 125b, 151, 335, and 365) thus demonstrating the involvement of miRNAs in T1D patients’ Treg cells [160].

## Benefits and risk of epigenetic therapy

As we have seen in the previous sections, mainly old well-studied and often already approved epigenetic modulators have been used in the context of autoimmune diseases [56, 59, 67, 81, 84, 86, 89, 103, 121, 159]. These first-generation molecules have been originally developed as anti-tumor therapy. They often show heavy side effects by mechanism, which are tolerable in cancer, but not in other diseases [9, 19]. This point is one major drawback and risk of the studies currently present in literature. The main challenge for researchers is not only to understand better the role of epigenetics in autoimmune disorders also with the help of epigenetic modulators, but also to develop novel specific targeted therapies. Interestingly, novel second or third generation epigenetic modulators, which often have a better selectivity and safety profile, have been used in other medical conditions but have not yet been applied in immunotherapy approaches [18, 161]. In our view, benefits outweigh the risks as novel innovative treatments are needed, but researchers should proceed with care considering the selectivity and safety profile of their potential treatments already at an early stage of the therapy development.

## Conclusions

This work has been focused on epigenetic mechanisms regulating several autoimmune disorders with the aim to provide new therapeutic ideas in this area of interest (Fig. 1). The link between epigenetics and autoimmunity has been widely documented in the literature; although, more and more extensive studies are required to better understand the influence of these modifications in the different autoimmune disorders. A deeper exploration of the complex epigenetic interactions may be useful for the development of promising treatment strategies targeting the epigenome. The fundamental work of Farh et al. in 2015 described a fine-mapping algorithm to identify non-coding genetic variants that could underlie autoimmune diseases from genotyping data [162]. However, to date, there is still a lot of research necessary in order to provide effective healthcare solutions for such diseases, such as an adequate choice of treatment and a precise prediction of treatment outcomes. Epigenetics will very likely aid to provide further progress in the field of autoimmunity. Until now, only a few studies have been carried out to examine the clinical applicability of epigenetics and its modulators in such autoimmune diseases. Keeping this concept in mind, more comprehensive and more accessible technologies are recommended to promote advances in the therapeutics leading to prevention by early interventions and reducing both costs and patient morbidity. Taken together, the future of epigenetics in autoimmunity is rapidly increasing. However, it still needs an in-depth investigation to allow novel potential therapeutic opportunities, better than those currently used.

## Abbreviations

AITD: Autoimmune thyroid diseases
DNMTs: DNA methyltransferases
GD: Graves’ disease
GEO: Gene Expression Omnibus
HATs: Histone acetyltransferases
HDACi: Histone deacetylases inhibitors
HDACs: Histone deacetylases
HT: Hashimoto thyroiditis
ICAM-1: Intracellular adhesion molecule-1
IF: Immunofluorescence
IRF-7: Interferon regulator factor 7
LincRNAs: Long intergenic noncoding RNAs
LncRNAs: Long noncoding RNAs
LRA: Largazole
miRNAs: microRNAs
MTRR: Methionine synthase reductase
OA: Osteoarthritis
PBMC: Peripheral blood mononuclear cells
PP2Ac: Protein phosphatase 2A
PRF1: Perforin
RA: Rheumatoid arthritis
RASF: Rheumatoid arthritis synovial fibroblasts
RFX1: Regulatory factor X1
SAM: S-adenosyl-methionine
SGEC: Salivary gland
SLE: Systemic lupus erythematosus
SS: Sjogren’s syndrome
SSc: Systemic sclerosis
T1D: Type 1 diabetes
TLR9: Toll-like receptor 9
TSA: Trichostatin A
VCAM-1: Vascular adhesion molecule-1
XCI: X chromosome inactivation
